# Can a Taste for Poison Drive Speciation?

**DOI:** 10.1371/journal.pbio.0050140

**Published:** 2007-04-24

**Authors:** Liza Gross

The endless struggle for survival in nature inevitably boils down to finding food and eluding predators. To avoid the latter, many plants produce chemical weapons to discourage predators. A sound strategy overall, but the rules of co-evolutionary war suggest that an herbivore will evolve resistance to the toxic defenses of plants.

The fruit fly*Drosophila sechellia*, for example, has a penchant for the fruit of a Polynesian shrub called Tahitian Noni*(Morinda citrifolia)*that smells so foul it’s nicknamed “vomit fruit.” Other*Drosophila*species treat the rank odor, which arises from the toxins hexanoic acid and octanoic acid, as a warning sign to stay away. And with good reason—if they alight on Tahitian Noni’s fruit, they die. But*D. sechellia*blithely homes in on the malodorous fruit to lay its eggs, ensuring a bounteous meal for its larval offspring.

*D. sechellia*’s resistance to a plant that kills likely competitors gives the fly nearly exclusive access to its host—a distinct ecological advantage. But it also raises an important question for evolutionary biologists: are the factors that promote specialized ecological interactions between herbivore and plant host sufficient to drive herbivore speciation? A group of researchers at Tokyo Metropolitan University were especially interested in learning how an ancestral population of flies acquired the ability to use a toxic plant as its breeding grounds.

In a new study, Takashi Matsuo, Yoshiaki Fuyama, and colleagues explored the genetic factors underlying the behavioral differences between*D. sechellia*and other*Drosophila*species. Taking advantage of the robust genetic tools offered by*D. melanogaster*, the researchers traced the flies’ divergent host-plant preferences to two olfactory genes,*odorant-binding protein 57e (Obp57e)*and*Obp57d*. Their findings suggest that as the expression patterns of these genes changed in*D. sechellia*, the fly lost the impulse to avoid Tahitian Noni, allowing an adaptive shift to this previously proscribed plant.

In a previous study, the researchers (Higa and Fuyama) had identified the genomic locus (on the second chromosome) responsible for*D. sechellia*’s attraction to hexanoic acid and*D. simulans*’ avoidance. In this study, they generated a higher-resolution map of that area by using a series of*D. melanogaster*mutant fly stocks, each missing a different part of the chromosomal region. After crossing these “deficiency strains” with*D. sechellia*, they determined which strains sired offspring with a preference for hexanoic acid and found a missing stretch of base pairs in an olfactory gene,*Obp57e*, that could be responsible for the altered behavior. Since the*D. sechellia Obp57e*gene didn’t have any mutations that would block its function, they concluded that the gene must be expressed differently in the fly.

**Figure:**
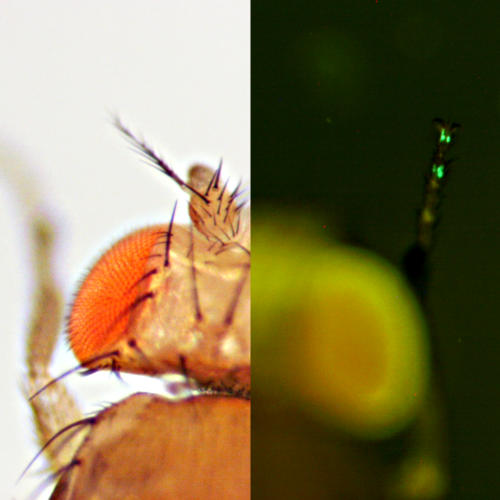
Genes expressed at the tip of fruit flies' legs determine which host plant they choose as their breeding grounds. (Image: T. Matsuo and J. Yasukawa)

When they measured*Obp57e*transcript levels in the three species, they found the highest levels in*D. sechellia*. In*D. melanogaster*,*Obp57e*has a characteristic, limited expression pattern driven by a short stretch of DNA called a promoter. The researchers cloned the corresponding sequence from*D. sechellia*and*D. simulans*and introduced it into different*D. melanogaster*stocks. The*simulans*sequence reproduced the initial*melanogaster*expression pattern, but the*sechellia*sequence, which contained four additional base pairs, did not. Removing the base pairs restored*Obp57e*expression in*melanogaster*, showing that they alter the gene’s expression in*melanogaster*—but what controls*Obp57e*expression in*D. sechellia*?

**Figure:**
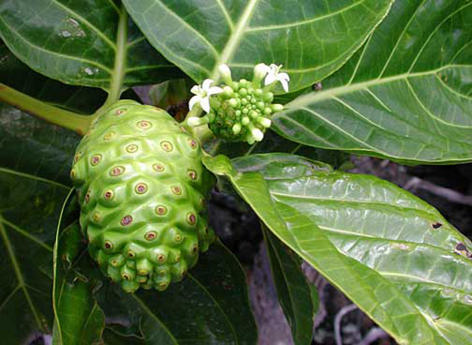
Morinda citrifolia. (Photo: NPS/Bryan Harry)

To find out, the researchers generated lines of “knock-out” flies that lacked either*Obp57e*genes, adjacent*Obp57d*genes, or both (called double knock-outs). Flies lacking just one of the genes avoided hexanoic acid–laden traps, whereas females missing both genes flocked to them. But the most interesting results came when the researchers compared the knock-out strains’ choice of hexanoic acid or octanoic acid as an egg-laying medium. When the*D. melanogaster*double knock-out received either the*D. sechellia*or*D. simulans*’ versions of*Obp57e*and*Obp57d*, it adopted the behavior of the donor fly. Thus, replacing*Obp57d*and*Obp57e*genes changed the fly’s response to the host toxins. The researchers conclude that an alteration in the expression pattern of the two genes produces this behavioral shift.

In future experiments, the researchers plan to minimize the interaction of these two genes to understand their separate functions. Until then, it appears that*D. sechellia*’s choice of forbidden fruit as a reproductive site involved genetic changes that promoted resistance to octanoic acid and transformed an urge to avoid the toxin into a fondness for its fetor. The researchers suspect that the fly lost its urge to avoid the fruit first; a plausible scenario if an ancestral population of flies landed on fruit in advanced stages of decay, when octanoic acid toxins have mostly degraded. Behavioral adaptations between herbivores and their hosts tend to involve changes in genes linked to taste and odor perception. With over 50*Obp*genes in the*D. melanogaster*genome, researchers have a rich resource for studying the ecological contributions to speciation.

